# Virus Prospecting in Crickets—Discovery and Strain Divergence of a Novel Iflavirus in Wild and Cultivated *Acheta domesticus*

**DOI:** 10.3390/v13030364

**Published:** 2021-02-25

**Authors:** Joachim R. de Miranda, Fredrik Granberg, Piero Onorati, Anna Jansson, Åsa Berggren

**Affiliations:** 1 Department of Ecology, Swedish University of Agricultural Sciences, 756 51 Uppsala, Sweden; piero.onorati@slu.se (P.O.); asa.berggren@slu.se (Å.B.); 2 Department of Biomedical Sciences and Veterinary Public Health, Swedish University of Agricultural Sciences, 756 51 Uppsala, Sweden; fredrik.granberg@slu.se; 3 Department of Anatomy, Physiology and Biochemistry, Swedish University of Agricultural Sciences, 756 51 Uppsala, Sweden; anna.jansson@slu.se

**Keywords:** metagenome, virome, *Acheta domesticus*, cricket, Acheta domesticus Iflavirus, strain evolution, cricket rearing

## Abstract

Orthopteran insects have high reproductive rates leading to boom-bust population dynamics with high local densities that are ideal for short, episodic disease epidemics. Viruses are particularly well suited for such host population dynamics, due to their supreme ability to adapt to changing transmission criteria. However, very little is known about the viruses of Orthopteran insects. Since Orthopterans are increasingly reared commercially, for animal feed and human consumption, there is a risk that viruses naturally associated with these insects can adapt to commercial rearing conditions, and cause disease. We therefore explored the virome of the house cricket *Acheta domesticus*, which is both part of the natural Swedish landscape and reared commercially for the pet feed market. Only 1% of the faecal RNA and DNA from wild-caught *A. domesticus* consisted of viruses. These included both known and novel viruses associated with crickets/insects, their bacterial-fungal microbiome, or their plant food. Relatively abundant among these viral Operational Taxonomic Units (OTUs) was a novel Iflavirus, tentatively named Acheta domesticus Iflavirus (AdIV). Quantitative analyses showed that AdIV was also abundant in frass and insect samples from commercially reared crickets. Interestingly, the wild and commercial AdIV strains had short, extremely divergent variation hotspots throughout the genome, which may indicate specific adaptation to their hosts’ distinct rearing environments.

## 1. Introduction

Historically, viruses were first identified through their association with disease symptoms, and only subsequently described genetically. This process is reversed in modern virus prospecting, where the viral agents are first identified and described genetically [1,2], and only subsequently investigated for possible biological roles or disease associations [3,4]. This has led to the realization that virus diversity is chronically underrepresented in the public nucleotide databases and that the vast majority of viruses are largely asymptomatic, with pathological viruses the exception rather than the rule [5]. However, this does not mean that such asymptomatic viruses cannot become pathogenic, under the right circumstances. A classic example of this is the deformed wing virus, which is currently the most common and lethal virus disease of honeybees worldwide [6,7], but was practically undetectable until it became associated with a new and highly potent transmission route, the ectoparasitic mite *Varroa destructor* [8,9]. This change in transmission route, pathological profile, and virulence proceeded through typical evolutionary virology stages and mechanisms [10,11,12], involving the evolution of distinct derivative strains optimized to the new transmission route [13,14], the co-circulation and spread of pre- and post-adapted strains through a common quasi-species [15] and the management of viral pathology, virulence, and quasi-species composition through control of the new transmission route [9,14,15]. What is particularly revealing from these, and other, studies is how closely deformed wing virus pathology is linked to a particular transmission context, and not others [7], and how potent this transmission context is for modulating virus virulence, both up and down [9,14,15]. 

Viruses, particularly those belonging to the Iflaviridae and Dicistroviridae [16,17], account for many of the diseases affecting mass-reared insects in historical industries, such as apiculture and sericulture [18,19]. This is partly because viruses are particularly well suited to the natural boom-bust population dynamics and high local and temporary densities of many insect species. These are important considerations for the emerging insect-as-food-and-feed industry [20,21,22], which aims to produce large volumes of healthy insects in the least amount of time and space. These are the ideal criteria for virus transmission, virulence evolution and disease development [10] and one of the reasons why viruses have been identified as a particular threat to the industry [23]. Disease evolution is a particular risk for those viruses with a major oral transmission route, such as the Iflaviridae and Dicistroviridae, in insect species with a propensity for cannibalism and coprophagy, such as crickets and many social insects. 

Orthopteran insects (grasshoppers and crickets) currently account for about 50% of the edible insects market by volume [24,25,26]. The two main cultivated cricket species are the house cricket (*Acheta domesticus*) and the two-spotted cricket (*Gryllus bimaculatus*), which are also projected to become cultivated for human consumption [27]. Practically nothing is known about the viruses infecting these crickets, other than the few already known to cause disease [22], despite the pressing need for knowledge about existing and potential new diseases and for management protocols to rear Orthopteran insects to acceptable animal welfare and food-feed health and safety standards [21,22]. In this study, we therefore explored the natural virome of wild house crickets, identified a novel and abundant Iflavirus, and characterized and compared two distinct strains of this virus in wild and commercially reared cricket samples.

## 2. Materials and Methods

### 2.1. Study Design

The study design consisted of an initial target-free screening of both the DNA and RNA phases of a single frass sample of wild-caught Swedish house crickets, followed by targeted screening, full genome assembly, and genomic comparison of two distinct strains of a novel Iflavirus isolated from wild and commercially reared *A. domesticus*.

### 2.2. Sample Origin and Processing

Wild Swedish house crickets were collected locally around Uppsala, Sweden. The individuals were kept in clean cages from where frass was obtained for analysis. Samples of live commercially reared house crickets (*A. domesticus*) and two-spotted crickets (*Gryllus bimaculatus*) and their frass were obtained from six different Swedish retailers, identified anonymously by the letters A–F (Table 1). Insect homogenates were prepared by pulverizing flash-frozen insects in BioReba meshbags (Bioreba, Reinach, Switzerland) with a pestle and resuspending in 2 mL sterile water per insect. Frass homogenates were prepared in 0.5 mL sterile water per 0.1 g frass using a MixerMill 400 beadmill (Retsch Haan, Germany) and ten 3 mm glass beads shaking at maximum speed for 60 s [28]. DNA was extracted from 100 µL homogenate using the Qiagen Blood and Tissue kit (Qiagen, Hilden, Germany) following the ‘Tissues and Rodent tails’ protocol and eluting the DNA in 200 µL AE buffer. RNA was extracted from a separate 100 µL aliquot of homogenate using the Qiagen RNeasy Plant Mini kit, following the ‘Plant’ protocol and eluting the RNA in 50 µL RNase Free water. The DNA/RNA concentration was estimated using a NanoDrop 1000 instrument (NanoDrop, USA), after which the samples were stored at −20 °C until further use.

### 2.3. Target-Free Screening by Sequencing

Around 1,0 μg DNA from the frass of wild-caught *A. domesticus* was submitted for PacBio sequencing by LifeSciLab in Uppsala, Sweden, in a single barcoded sequencing run together with honeybee DNA samples. The PacBio-reads were demultiplexed and delivered as circular consensus sequencing (CCS) reads in FASTQ format. Around 1.5 μg of total RNA extracted from the same frass sample was submitted for Ion Proton S5XL sequencing by LifeSciLab in Uppsala, Sweden. Ribosomal RNA was removed from the sample using the Illumina RiboZero rRNA depletion kit. The remaining RNA was quantified and checked for integrity using the Agilent Bioanalyzer and the sequencing library was constructed using the Ion Total RNA-Seq v2 kit. The library was barcoded and included in a single sequencing run with similarly barcoded RNA sequencing libraries from bumblebees and Indian tasar silkworms. Sequencing was performed on an Ion S5XL instrument using an IonChef S5 530 chip. The reads were delivered demultiplexed in BAM files and converted to FASTQ format using the SamToFastq tool in the Picard package v2.23.4 [29]. Both the Ion S5XL and PacBio reads were trimmed and passed through quality control using FastQC [30] and the Fastx-Toolkit [31]. Taxonomic assignment was performed by comparing reads against a local copy of the NCBI nr database (downloaded on 3 June 2020) using DIAMOND BLASTx v0.9.31 [32]. The resulting DAA files were imported into MEGAN6 v 6.19.7 [33] using the following mapping file: megan-map-Jul2020-2.db. The quantitative and phylogenetic distributions were also visualized using hierarchical pie charts produced with Krona Tools v2.7 [34]. The taxonomic data were evaluated for potential viral pathogens and candidate reference genomes were identified and retrieved from GenBank in FASTA format. The reads were assembled into contigs using both SPAdes v3.11.1 [35] and MegaHit v1.1.2 [36] with default settings. These contigs were imported into CodonCode Aligner v8.0.1 (CodonCode Corporation, Dedham, MA, USA) and Geneious Prime v2020.2.4 (Biomatters Ltd., Auckland, New Zealand; [37]) in order to assess the contig quality, merge partially overlapping contigs, and compare with candidate reference genomes. The Geneious platform was also used to organize the contigs for each sample into BLAST databases against which potential reference sequences could be compared on amino acid level using the tBLASTx algorithm. The DNA sequencing yielded only 788 reads with a mean length of 942 bp, of which about 83% were estimated to be unique. Taxonomic identities were assigned to 783 of the reads, but no viruses were identified. The RNA sequencing yielded a total of 2,322,040 raw reads with a mean length of 93 nt, of which 37.5% were estimated to be unique and 2,221,590 (95.7%) were retained for analysis after quality filtering. Taxonomic identities were assigned to 445,000 of the reads, of which about 10 % (44,197 reads) could not be assigned to any metagenomic taxa (Figure S1). The assembly of the RNA reads resulted in 491 contigs, between 3435 and 129 nt long, using SPAdes and 3184 contigs, between 3742 and 200 nt long, using MegaHit. Among the reads classified as viral, the great majority (4047) belonged to the order Picornavirales. Closer analyses of the reads and the contigs indicated that they had global similarity with existing viruses within this order. All assembled contigs (generated with both Spades and MegaHit) were therefore used for a second assembly, which succeeded to join some of the contigs into longer scaffolds. However, complete viral sequences were not obtained. For the novel Iflavirus, both contigs and scaffolds were compared at amino acid level with Perth bee virus 3 (Table S3), the closest publicly available reference genome.

### 2.4. Full Genomes of Wild and Reared AdIV Strains by Sanger Sequencing

The incomplete assembled AdIV proto-sequence was used to design RT-PCR assays spanning the entire genome (Table S1). The full genome sequences of two strains of the new Iflavirus were obtained by Sanger sequencing (Macrogen Europe BV, Amsterdam, The Netherlands) these RT-PCR amplicons from frass samples of wild-caught and commercially reared *A. domesticus* (Table 1). The 684 nt helicase fragment generated with the AdIV-HeHel-F/R primer pair (Table S1) was Sanger sequenced for all positive samples, in order to identify the dominant strain in these samples. The nucleotide differences between the wild and commercial AdIV strains were analysed for the proportion of transition changes (A-G and C-T), the overall frequency of nucleotide substitutions, and the frequency of non-synonymous substitutions within a moving 90-nucleotide window across the genome.

### 2.5. Phylogenetic Analyses

The polyprotein amino acid sequences of the new Acheta domesticus Iflavirus (AdIV) were aligned to a selection of 23 related Iflaviruses (Table S2) by CLUSTAL-W, as implemented by MEGA-X [38], using the default gap creation and extension penalties. The complete amino acid sequence of the Iflavirus polyprotein was used for phylogenetic analysis. All positions containing gaps or missing data in the multiple alignments were excluded from the phylogenetic analyses, resulting in a total of 1946 characters (amino acids) for the analysis. The phylogenetic relationship between the different taxa was inferred using the Maximum Likelihood method and our default JTT matrix-based amino acid substitution model [39]. Several other substitution models offered in MEGA (*e.g.* Dayhoff, Poisson, Equal Input, WAG, LG) were also tried but these made little difference to the likelihood estimate, tree shape and composition, or the confidence in the branching pattern. Initial tree(s) for the heuristic search were obtained automatically by applying Neighbor-Join and BioNJ algorithms to a matrix of pairwise distances estimated using the JTT model, and then selecting the topology with superior log likelihood value. The phylogenetic tree with the highest likelihood was retained. The statistical confidence for each branching node was determined by bootstrapping the alignment 500 times, to calculate the percentage of bootstrapped trees retaining the taxa clustered by each node in the most likely tree.

### 2.6. Targeted Screening for AdIV by RT-qPCR

The presence and amount of the novel Acheta domesticus Iflavirus was determined by quantitative RT-qPCR. The RNA of each sample was first converted to cDNA using the InVitrogen SuperScript-III 1st-strand cDNA kits (ThermoFisher Scientific, Waltham, MA, USA) and diluted 5-fold in ultrapure water. The cDNA was then used as template for qPCR, using forward and reverse qPCR assay designed in a highly conserved section of the Iflavirus RNA-dependent RNA polymerase region (Table S2) to be compatible with the thermocycling profile for the AdDV VP gene [28], consisting of initial denaturation for 120 sec at 98 °C followed by 40 cycles of: denaturation for 10 sec at 98 °C, annealing-extension for 30 sec at 58 °C and optical reading. This was followed by a Melting Curve analysis to validate the PCR product identity, by reading the fluorescence at 0.5 °C increments from 65 °C to 95 °C. All assays were run in duplicate using the SsoFast EvaGreen Svupermix kits (BioRad, Hercules, CA, USA) with the mean Cq value used for quantitative analyses [40]. Only data from the first 35 cycles were used for making detection assessments, due to the risk of false positive or false negative results beyond 35 cycles of amplification [41].

## 3. Results

### 3.1. Target-Free Screening of Acheta Domesticus Frass DNA and RNA

**Figure 1:**
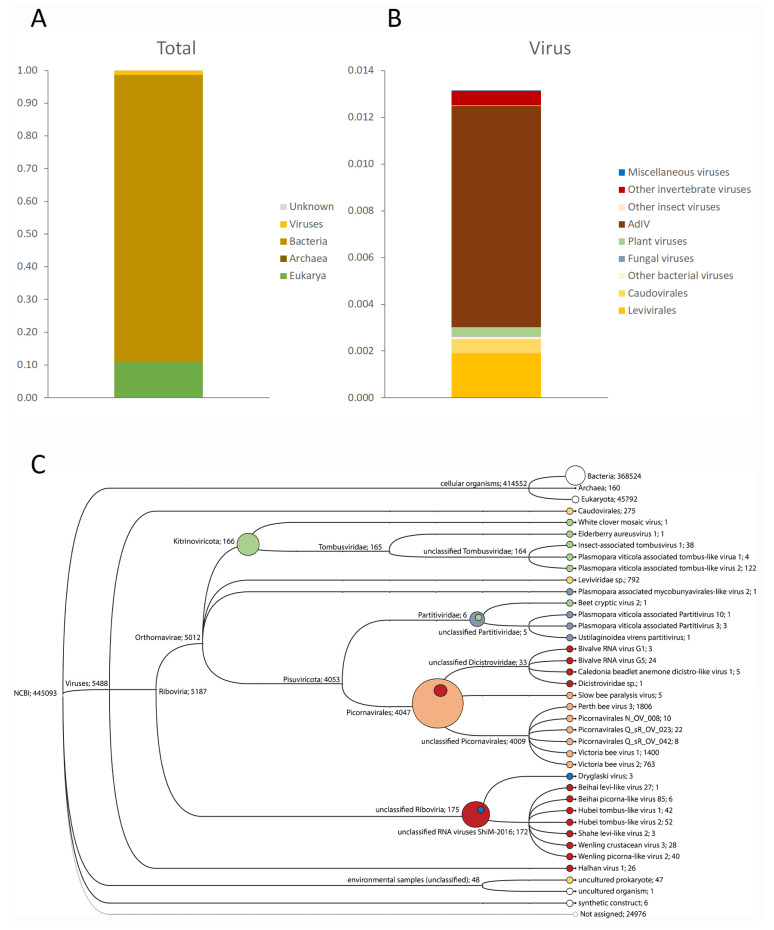
Taxonomic composition of *Acheta domesticus* frass. (**A**) Full composition of the wild *Acheta domesticus* frass sample at the level of Domain (Archaea, Bacteria, Eukarya, and Viruses) as determined by RNA sequencing. (**B**) Composition of the 1% virus subfraction, separated by primary host: bacteria (yellow), fungi (grey), plants (green), insects (orange), invertebrates (red), and miscellaneous viruses (blue) whose host status is unclear. (**C**) Cladogram of the viral reads obtained through RNA sequencing, with the primary hosts of the virus identified by coloured dots, as for Figure 1B. The composite circles represent the host distribution at higher virus taxonomic categories. The numbers indicate the number of unique reads assigned to the corresponding taxon.

Insufficient PacBio DNA sequencing reads (788) were obtained for any meaningful metagenomic analyses. The taxonomic assignments of these reads were split evenly between plants (mostly wheat and barley), and a selection of gut bacteria, mostly *Proteobacteria*, *Firmicutes,* and *Bacteriodetes* (Figure S1A). No viral DNA sequences were identified. The same bacterial and plant taxa were also recovered by the IonProton RNA sequencing, but in different proportions, with about 10% assigned to plants, 88% to bacteria, and 1% to viruses (Figure 1A; Figure S1B). These viral reads were assigned to a wide range of taxa spanning the full breadth of virus taxonomy (Figure 1C), with mostly bacterial, insect, and plant hosts (Figure 1B). The single most abundant group of virus reads were originally assigned to three closely related Iflaviruses: Perth bee virus-3, Victoria bee virus-1, and Victoria bee virus-2 (Figure 1C) [2], but were subsequently shown to belong to a single, novel Iflavirus, tentatively named Acheta domesticus Iflavirus (Figure 1B). Most of the remaining viral reads were assigned to two viral Orders specific to bacteria: *Caudovirales* (bacteriophages) and *Levivirales*. A minor fraction of reads were assigned to various members of the plant-infecting virus Family *Tombusviridae*, and to various viruses identified through a massive virus prospecting study of a wide range of invertebrates in China [1].

The novel Acheta domesticus Iflavirus (AdIV) was also frequently detected in a range of *Acheta domesticus* insect and frass samples obtained from several commercial cricket rearers and retailers, but not in a batch of *Gryllus bimaculatus* crickets and frass (Table 1).

### 3.2. Genetic Analysis of AdIV Strains from Wild and Commercially Reared Crickets

**Figure 2:**
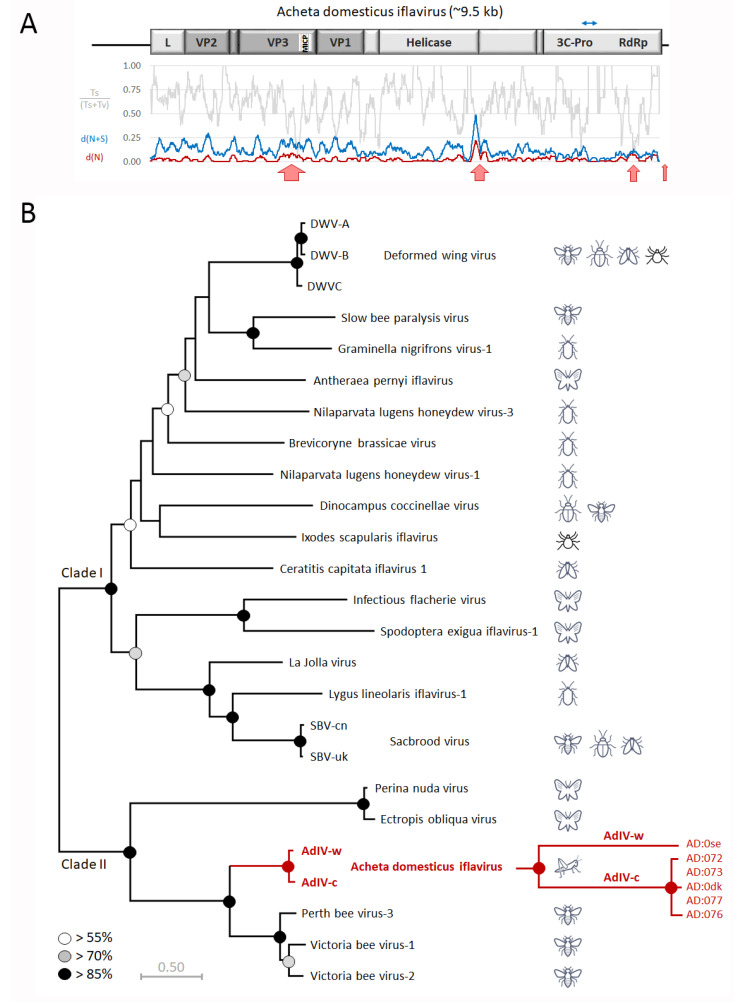
Genome organization and phylogenetic placement of Acheta domesticus Iflavirus. (**A**) Genome organization of Acheta domesticus Iflavirus (AdIV), showing the location and size of the structural (VP2, VP4, VP3, VP1; dark grey) and non-structural (L protein, helicase, 3C-protease, and RNA dependent RNA polymerase; light grey) genes, separated by the location of putative 3C-protease cleavage sites. The chart below the genome map summarizes the nature of the nucleotide differences between the two strains in a moving 90-nucleotide window for: the proportion of all changes that are transitions (Ts/(Ts+Tv): grey); the overall rate of synonymous and non-synonymous change, (d(N+S): blue), and the rate of non-synonymous change, (d(N): red). The blue arrows identify the location and size of the AdIV diagnostic RT-qPCR assay. The red arrows identify the location and size of the high variability hotspots. (**B**) Location of the wild (AdIV-w) and commercial (AdIV-c) strains of AdIV (red branch) in the Iflavirus phylogenetic tree, based on analysis of the full polyprotein amino acid sequences of a representative selection of Iflaviruses (Table S2). The insect icons indicate the Families of their primary hosts. The tree is draw to scale, measured in amino acid substitutions per site. Also included is a high-resolution phylogeny of the six positive samples (Table 1), based on 684 nucleotides in the helicase region. Groups of taxa that cluster significantly are identified by the white, grey, and black (red) circles on the respective branching nodes.

The full genome sequences of two strains of the new Iflavirus, obtained from the frass samples of wild and commercially reared *A. domesticus* respectively, were obtained by Sanger sequencing PCR amplicons spanning the entire proto-sequence assembled from the IonProton data. Acheta domesticus Iflavirus has a typical Iflavirus genome organization (Figure 2A), consisting of a single positive-stranded RNA of about 9–10 kb coding for a single polyprotein bounded on the 5′ and 3′ end with non-translated regulatory regions (NTR) and terminating in a natural poly-A tail [16]. The virus structural proteins (VP1-VP4) are located in the N-terminal half of the polyprotein, preceded by a short leader (L) protein, while the non-structural proteins, principally the helicase, a 3C-protease and RNA-dependent RNA polymerase, are located in the C-terminal half of the polyprotein, which is also highly typical for just Iflaviruses [16].

The two AdIV strains are ~90% identical at nucleotide level, with most of the variation located in redundant codon positions, resulting in ~93% amino acid identity across the polyprotein. However, the variation is unevenly distributed across the genome, with multiple variability hotspots, especially in the structural protein region (Figure 2A, blue line). Across the genome there is a direct relationship between the proportion of these substitutions that are transversions (Figure 2A, grey line) and whether or not they also result in amino acid changes (Figure 2A, red line). Transversion substitutions are more likely to result in amino acid changes than transition substitutions, due to the nature of the redundancy in the triplet codon [42]. This is particularly evident in three hotspots: in a relatively broad region of amino acid change just prior to the P-domain (MICP) of the VP3 structural protein; in a short region of high nucleotide and amino acid change close to a predicted helicase protease cleavage site, and short section of the RNA-dependent RNA polymerase with a high degree of amino acid variability relative to the overall nucleotide variability (Figure 2A, red arrows). The variability in the VP3 and helicase hotspots have relatively average ratios of synonymous vs. non-synonymous changes, while nearly all of the nucleotide changes in the polymerase hotspot are non-synonymous.

The hotspot in the helicase is generated by several frame-shifts, caused by small insertions and deletions (indels), which also results in the net gain of two amino acids for the commercial strain, relative to the wild strain. The final major difference between the two strains is in the 3′ Non-Translated Region (3′NTR), which for most Iflaviruses is about 300 nucleotides long, but much shorter (46 nucleotides) for the commercial strain and only 17 nucleotides for the wild strain.

Phylogenetic analysis of the AdIV amino acid sequences locates the virus on its own branch in a very well resolved part of the Iflavirus phylogeny (clade II; Figure 2B), unambiguously separated from its nearest relatives, a group of three viruses identified in Australian honeybees [2]. The topology of the phylogeny is very similar to those obtained for individual regions of the polyprotein (VP1-VP4; Helicase; 3C-RdRp region), particularly for clade II where AdIV is located. Only in clade I of the phylogeny do the taxa affiliate differently according to the genome region analysed, as indicated by the poor bootstrap support for many of the branching nodes in this sector. The differences between the two AdIV strains are of a similar magnitude as for the major strains of deformed wing virus and sacbrood virus, and for the difference between Ectropa obliqua virus and Perina nuda virus. A high-resolution analysis of a 684 nucleotide segment of the helicase gene showed that all the AdIV-positive commercial cricket samples contained the same AdIV-c strain, while the AdIV-w strain was only detected in the wild cricket frass sample.

## 4. Discussion

Target-free screening of DNA and RNA samples is a hugely powerful tool for a comprehensive description of the complexity and diversity of microbiomes, including viromes. It is based on two recent developments in molecular biology: cheap high throughout mass parallel sequencing and extensive public nucleotide sequence databases, against which the new sequences are compared for making a taxonomic identification. If the match is perfect, a previously described organism has been detected. If the match is made at a higher taxonomic level, then a new member of that taxon has been identified. As a consequence, target-free screening of microbial samples is also hugely powerful for discovering new microbial taxa, ranging from new Species and Genera all the way to new Families and even new Orders [1,2]. The rate at which these new discoveries are made is a reflection both of the diversity of the microbial world and of the inadequate cataloguing of this diversity by the nucleotide databases, whose content is heavily biased towards those (few) microorganisms directly relevant to human progress. The current study is a typical example of this approach. About half of the Iflaviruses included in the phylogeny were first identified through disease symptoms and the other, more recent, half through RNA sequencing. The initial identification of AdIV was indirect, through similarity at amino acid level to three closely related reference viruses. It was only through closer examination of these three separate identifications that it became apparent that they all related to a single, novel virus, with different parts of the genome identifying differently to the three reference viruses. Similar partial, uneven, and split assignments may very well also exist for some of the other viruses identified, particularly the bacteria-infecting viruses (Caudovirales, Levivirales), and for the bacterial microbiome in general.

Although identifying a new virus has become rather easy, confirming the host status of the organism it was first detected in remains more complicated. Koch’s postulates [43] were originally conceived for proving the causality of the symptoms associated with pathogenic microorganisms, which exclude the vast majority of microorganisms with no, unclear, or chronic symptoms: something that Koch himself already realized [3]. Koch’s postulates have therefore been periodically adapted to incorporate new knowledge and technology, particularly the ability to detect and identify microorganisms simply through analysing nucleic acids, as is the case here. Similar adaptations have also been made to related concepts, such as virulence, infectivity, pathogenicity and symptomatology [4]. Through these, the main criteria for establishing the host’s status are evidence that the microbial agent replicates inside the host tissues. Since the replication-transcription intermediates frequently used to establish host-status for single-stranded RNA viruses [44,45] are not available in frass samples, where AdIV was first identified, the main evidence that *Acheta domesticus* is indeed a host for AdIV is the sheer volume of RNA reads assigned to the virus. Iflaviruses are insect-specific, so the only two explanations for the high volume of AdIV reads in cricket frass is either that *Acheta domesticus* is a host, shedding large volumes of AdIV propagules into the gut lumen, or that the virus is acquired passively, through the consumption of infected insects. However, no RNA reads were recovered from the frass sample identifying with an insect other than *Acheta domesticus*, which would be expected with passive acquisition. Moreover, large quantities of the virus were also recovered from unrelated *A. domesticus* frass and tissue samples. Therefore, the most likely explanation for the total data presented is that *A. domesticus* is a true host for AdIV. However, this conclusion must remain provisional until more conclusive evidence, *e.g.* from infection experiments, is obtained.

Neither the diversity nor the amount of virus detected is, in and of itself, unusual or alarming. Viruses are a natural part of life and the vast majority are asymptomatic, with no deleterious effects on their hosts [5]. Some are even beneficial or part of a symbiotic relationship [46]. The risk with viruses lies primarily in their capacity to adapt rapidly to changing circumstance, particularly those governing transmission [5,10,11]. These circumstances are very specific for each individual virus but the process can be extremely powerful, capable of transforming an insignificant, asymptomatic virus into a major pathogen. A well-studied example is deformed wing virus, whose pathological character is entirely linked to a new transmission route for which it was much better adapted than more established rival bee viruses [7,8]. This resulted in the evolution of several co-circulating major strains of the virus adapted to both the old and new transmission routes, allowing the virus to persist through multiple transmission routes [9,14,15]. The discovery of distinct strains of the new Iflavirus in wild and reared cricket samples may therefore be significant in this context, especially since Iflaviruses in general are highly adaptive and as a family overrepresented among disease-causing insect viruses.

Most of the genetic difference between the two AdIV strains involves nucleotide substitution: by far the most common means of introducing new genetic variation in RNA viruses [10,11]. The other types of genetic variation identified were insertions and deletions. No clear signs of recombination, inversion, or duplication were identified, although such processes are both common and expected in RNA viruses [10,11]. Even though transition substitutions (C-U and A-G changes) only account for four of the 12 possible nucleotide substitutions, they are by far the most common in RNA viruses since they derive in large part from the relative thermodynamic stability of the G-U pairing in RNA molecules during replication [47,48]. However, the overall proportion of transversion substitutions, which are more likely to result in amino acid change [42], is much higher for AdIV (~37%) than other Iflaviruses such as DWV and SBPV (both ~18%) [49,50]. Whatever the cause of this high ratio of transversions, which could be a property of the virus, the host or both, it makes it much easier for AdIV to generate amino acid variation for a given level of nucleotide variation. AdIV could therefore have a higher adaptive potential at amino acid level than other Iflaviruses.

The genetic differences between the two strains were unevenly distributed across the genome, in smaller and larger variation hotspots. Four of these hotspots were identified as particularly interesting, either because of the type genetic change, the concentration of non-synonymous changes and/or the genome location of the hotspot. One broad hotspot was located immediately prior to the P-domain of VP3 structural protein. The VP3 P-domain forms the protrusions on the Iflavirus virus particle that bind to the cell membrane receptors and mechanically inject the viral RNA into the cell for replication, and is therefore directly involved in the cellular infection process [51]. Variation in this region would affect the efficiency of this process, and perhaps the types of receptors, cells, and tissues infected. A second major hotspot was located in the helicase region of the polyprotein. This hotspot was generated by multiple small, frame-shifting insertions and deletions, which very efficiently generated major change in the amino acid sequence across this section, including the addition of two new amino acids to the commercial strain relative to the wild strain. The extreme divergence of this hotspot within an otherwise highly conserved section of the genome suggests that this may be a relatively recent change and still in the process of optimization. The helicase hotspot is located in a region where in DWV [49,52], SBPV [50], and SBV [53] there is a clear 3C-protease cleavage site between the C-terminus of the helicase and a peptide of unknown function between the helicase and the VPg, just prior to the 3C protease region (Figure 2A). It is unclear whether the variable region is part of the C-terminus of the helicase, in which case it is most likely responsible for the self-oligomerization of helicase peptides into a functional protein [54] or whether it is part of the N-terminus of a separate peptide of unknown function. The third hotspot concerns a high concentration of non-synonymous, transversion-dominated nucleotide substitutions in the ‘thumb’ domain of the RNA polymerase, spanning motifs D, E, and H. The thumb is the most genetically diverse of the three RNA polymerase domains (‘thumb’, ‘palm,’ and ‘fingers’) and is involved in binding the template during RNA replication, including binding the VPg protein that stabilizes the 5′ terminus of the RNA genome [55,56]. The ‘palm’ and ‘fingers’ domains are responsible for polymerization and nucleotide binding, respectively [56]. Variations in this region will affect the efficiency of template binding, which is the main factor controlling replication specificity and a strong contributor to replication efficiency [55,56]. The final hotspot of interest concerns a 29 nucleotide deletion in the already very short 3′NTR of the wild strain, leaving only 17 nucleotides between the polyprotein stop codon and the poly-A tail. The 3′NTR is normally highly conserved between major strains of Iflavirus species, much more so that the 5′NTR, and contains important regulatory signals for genome replication and translation [16], although such signals can also overlap with parts of the coding region. Major changes in this region can therefore be expected to affect replication efficiency.

The most common origin of natural genetic variation in viruses is biogeographic isolation, *i.e.* through the independent, relatively random, and evenly distributed divergence caused by physical (geographic) or biological (host range) barriers to the admixture, competition, selection, and genetic interchange between virus strains. The robust bootstrap support across the entire genome for the phylogenetic branch on which AdIV lies suggests that the virus has evolved in relative isolation and is not the result of recombination or horizontal gene transfer between different (Ifla)viruses [10,11]. However, the strong concentration of the variation between the two AdIV strains in a few hypervariable hotspots, as well as the type of variation and their location in key genomic regions directly involved in cell entry and replication efficiency suggests that this variation may have a different origin, more akin to the strain development seen for deformed wing virus and sacbrood virus. That both the wild and commercial AdIV-infected crickets appeared healthy is largely incidental to this process. Mortality, disease, or behavioural symptoms are not inevitable consequences of virus infection, but are rather adaptive properties that can be modulated to optimize survival at different scales of host organization and density [10,11]. If they facilitate virus transmission, they are retained. If not, they will be selected against. Since viruses need a living and reasonably healthy host in order to replicate, and since most known viruses are asymptomatic and, thus, presumably in a relatively benign relationship with their hosts, it seems that mortality and disease are not particularly important adaptive properties for viruses. Such attributes are more the product of specific circumstances than an innate necessity for the viral life cycle [4]. AdIV is so far the only Iflavirus identified from Orthoptera in a phylogeny dominated by viruses from Lepidopteran, Hemipteran, and Hymenopteran hosts, and by viruses with a gastrointestinal infection profile. Which, if any, of these two variants is ancestral and which is derived (*i.e.*, whether AdIV is natural and adapted to commercial rearing, or whether AdIV originated in commercial rearing, escaped, and adapted to natural cricket transmission) cannot be determined from these initial comparisons. Other major variants of the virus may well also exist, in different hosts or geographical locations, as has been shown for other Iflaviruses. These questions about origin, infectivity, and host range can only be answered by experimentally manipulating the transmission context for different AdIV strains under controlled conditions, and following the fate of the variability hotspots. These types of experiments are currently in progress.

## Figures and Tables

**Table 1:** Sample origins and Acheta domesticus Iflavirus (AdIV) status. Details of the samples analysed in the study, including the sample ID, the supplier of the samples (retailer or breeder), the date the sample was received, the species and developmental stage of the crickets, the sample type analysed, and the semi-quantitative results of the AdIV RT-qPCR test, with ‘-‘ indicating a negative result and ‘+’, ‘++’, and ‘+++’ indicating various levels of positive results. Samples AD:0se and AD:072 (**bold**) were used to obtain the full AdIV genome sequences of the two strains. For the other positive samples, the strain was identified by amplifying and sequencing a fragment of the helicase region.

SampleID	Supplier	Date	Species	Stage	Type	AdIV
**AD:0se**	**Wild**	**2017-09-26**	***Acheta domesticus***	**adult**	**frass**	**+++**
AD:0dk	A	2017-05-03	*Acheta domesticus*	adult	frass	+
AD:057	B	2020-01-22	*Gryllus bimaculatus*	adult	insect	-
AD:061	B	2020-01-22	*Gryllus bimaculatus*	adult	insect	-
AD:065	B	2020-01-22	*Gryllus bimaculatus*	adult	frass	-
AD:069	C	2020-06-28	*Acheta domesticus*	nymph	insect	-
**AD:072**	**C**	**2020-06-29**	***Acheta domesticus***	**nymph**	**frass**	**+++**
AD:073	C	2020-06-29	*Acheta domesticus*	juvenile	frass	++
AD:074	C	2020-06-29	*Acheta domesticus*	adult	frass	-
AD:075	D	2020-09-09	*Acheta domesticus*	adult	insect	-
AD:076	D	2020-09-09	*Acheta domesticus*	adult	insect	++
AD:077	D	2020-09-09	*Acheta domesticus*	adult	insect	++
AD:078	E	2020-09-25	*Acheta domesticus*	nymph	insect	-

## Data Availability

The PacBio and IonProton S5XL data presented in this study are openly available in the Small Read Archives (SRA) of the National Center for Biotechnology Information (NCBI: https://www.ncbi.nlm.nih.gov/) under accession numbers SRR13582029 and SRR13582030 respectively. The consensus genome sequences of the two strains of AdIV are available at GenBank (https://www.ncbi.nlm.nih.gov/genbank/) under accession numbers MW281483 and MW548506 for the wild-caught and commercially reared *A. domesticus* frass samples respectively.

## Supplementary Materials

The following are available online at https://www.mdpi.com/1999-4915/13/3/364/s1. Figure S1: Taxonomic composition of the frass metagenome [57], Table S1: RT-PCR assays, Table S2: Iflavirus host, discovery, and accession number [58,59,60,61,62,63,64,65,66,67,68,69,70,71,72].
