# Supplementary material for: Virus Prospecting in Crickets—Discovery and Strain Divergence of a Novel Iflavirus in Wild and Cultivated Acheta domesticus
Source: Viruses. 2021 Feb 25;13(3):364. doi: 10.3390/v13030364 (PMC7996529; doi:10.3390/v13030364)
Supplement: Supplementary file 1 [file viruses-13-00364-s001.pdf]

viruses

MDPI

Supplementary files

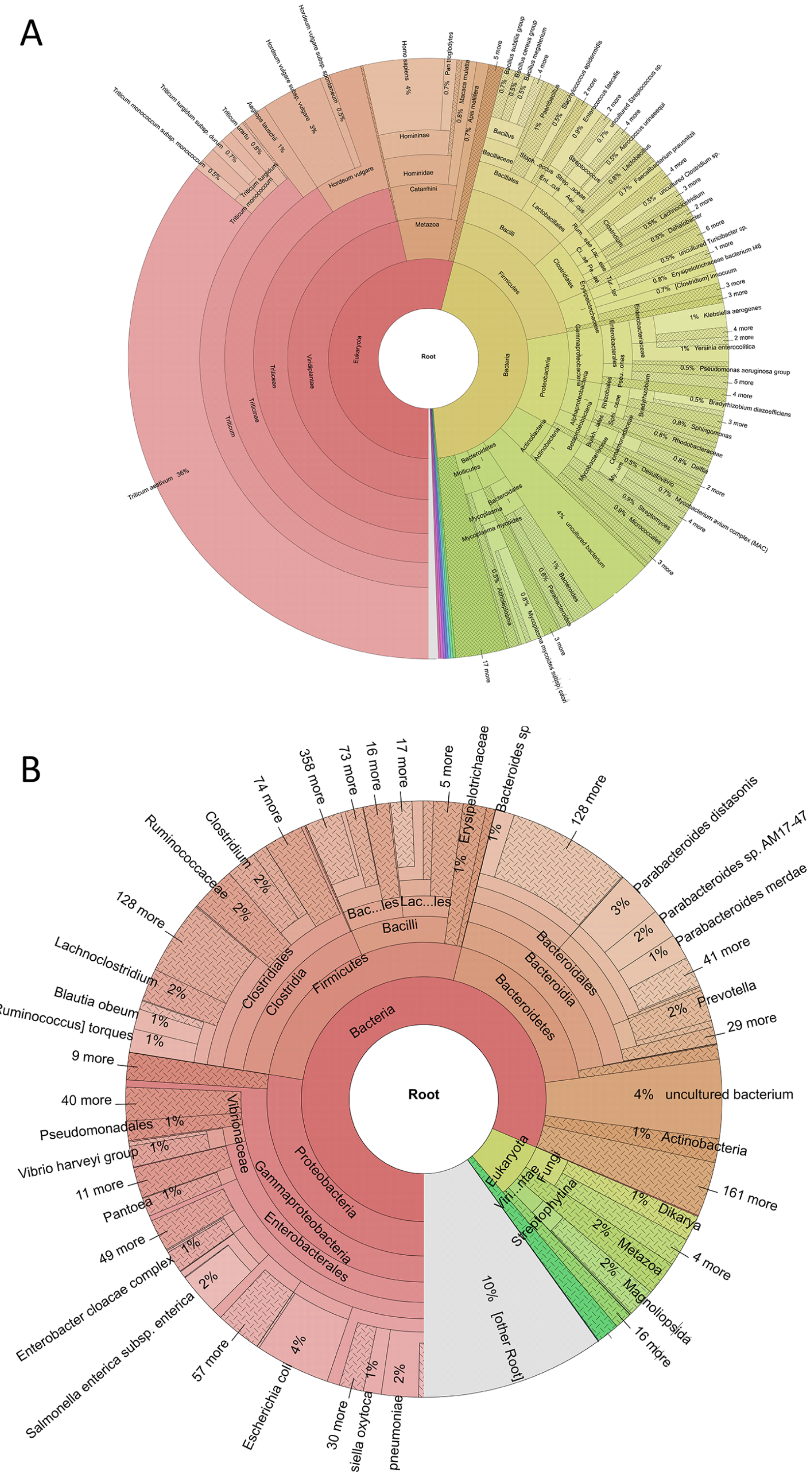

Figure S1. Krona charts.
Taxonomic distribution of (A) PacBio DNA sequencing reads and (B) IonProton RNA sequencing reads, as visualized by Krona Tools v2.7 [34]. About two-fifths of the bacterial reads in both the DNA and RNA sequencing were assigned to the Phylum Proteobacteria: 75% to a variety of Citrobacter species with the remaining 25% assigned to a diverse range of Enterobacterales, including a

number of well-known pathogenic taxa, such as Salmonella enterica, Escherichia coli, Klebsiella and Yersinia [57]. The next most abundant bacteria, accounting for about a fifth of the reads, were assigned to the Phylum Firmicutes: about 50% to the Order Clostridiales, 30% to Lactobacillales and 20% to Bacillales, each of which also includes a number of pathogenic taxa, such as Clostridium, Streptococcus, *Enterococcus faecium* and *Bacillus cereus*. The next most abundant bacteria, also accounting for about a fifth of the reads, were assigned to the Phylum Bacteroidetes: about 40% to the genus Bacteroides (including the pathogenic *B. fragilis*), 40% to other Bacteroidales and 20% to a variety of Flavobacteriales. The remaining fifth of bacterial reads were mostly assigned to the Order Fusobacteriales (primarily *Fusobacterium varium*) in the Phylum Fusobacteria, with a minor fraction going to a range of Actinobacteria.

Table S1. RT-PCR assays.

Details of the RT-PCR assays used in the study, including the genomic region targeted; the primer names and sequences and the PCR product size. These assays were used to obtain the consensus sequences of the complete genomes of the two strains of AdIV. Primers AdIF (F) and AdIF (R) (underlined) were also used for the quantitative RT-qPCR assays, producing a 212 nt amplicon. The 684 nt AdIV-HeHel assay was also used to obtain partial sequences for all positive samples.

Target	Primers	Sequence	Size
5' NTR	AdIV-5NTR (F)	CTTTCCGCAACGTTTTGCGTGTTTTGAG	788
	AdIV-5NTR (R)	ACAGTAAGGGCAGGTACAGA	
Lp	AdIV-Lp (F)	GCCTTCCTTCGTGTCCC	416
	AdIV-Lp (R)	GTTGATGAGTCAAGGGTGAA	
VP2	AdIV-VP2(F)	CAAAGAAGTGACCCAATCCTC	670
	AdIV-VP2 (R)	GCTAATTCGGGTCTCGCC	
VP4	AdIV-VP4 (F)	CCGGTGTCGCTAATTATGC	1166
	AdIV-VP4 (R)	GGTGACAATAGGATTGACAAG	
VP1	AdIV-VP1(F)	CTACCTATGCACCCATCTATTC	691
	AdIV-VP1 (R)	CCTAGTCGAGCTATGATGCC	
VP3	AdIV-VP3 (F)	AGTGCATGAACATAATCCTCC	1221
	AdIV-VP3 (R)	GAATGGTACTGGAGGGC	
Helicase	AdIV-VPHel (F)	TGATGATGTCCAACGAGCG	822
	AdIV-VPHel (R)	CATCCCAGTATTCTTGGCC	
Helicase	AdIV-HeHel (F)	CTCATCCCCTACCTCGC	684
	AdIV-HeHel (R)	GTCTACGGTTGCTTCTGG	
Helicase	AdIV-Hel (F)	GATGGTGAAGCAGAGGCG	685
	AdIV-Hel (R)	GGTCACAGAGATAGAAAAGGG	
3C-protease	AdIV-He3C (F)	GAAGGAGAAGTTTGTAGAAGAAG	811
	AdIV-He3C (R)	AGATCTCATATCAACTAGGGG	
3C-protease	AdIV-F (F)	CCATGAGAGACACTTCATCTG	476
	AdIV-3C (R)	CACATGATGGATGATGGTTGG	
RNA polymerase	AdIV-3C (F)	ACCACGGGTTCGAGGG	758
	AdIV-R (R)	CCGTTCATCTTTCAGAAAGAG	
RNA polymerase	AdIV-RdRp (F)	ATGGATCGACTGTTTGCCTG	501
	AdIV-RdRp (R)	CAACACATCACTCTTACCACG	
RNA polymerase	AdIV-F (F)	CCATGAGAGACACTTCATCTG	1052
	AdIV-RdRp (R)	CAACACATCACTCTTACCACG	
3' NTR	AdIV-3NTR (F)	GCTTGTACTTGGCTGCTC	373
	AdIV-3NTR (R)	TTTTTTTTTTTTTTTTTTTTTTTTTATAGC	

Table S2. Iflavirus host, discovery and accession number.
Details of the Iflavirus sequences used for the phylogenetic analyses, including the virus name, its primary host, how the virus was first discovered and its GenBank accession number, with the primary reference shown in square brackets.

Virus	Primary Host	Symptoms-Discovery	Accession [ref]
Acheta domesticus iflavirus	House cricket	RNA seq	MW281483<br>MW548506
Antheraea pernyi iflavirus	Chinese oak tasar silkworm	Larval vomiting disease	KF751885 [58]
Brevicoryne brassicae virus	Cabbage aphid	RNA seq	EF517277 [59]
Ceratitis capitata iflavirus-1	Mediterranean fruit fly	RNA seq	GAMC01001920 [60]
Deformed wing virus	Honeybee	Covert, deformities with injection	AJ489744 [49]<br>AY251269 [13]<br>CEND01000001 [61]
Dinocampus coccinellae paralysis virus	Spotted ladybird	Behaviour, parasitic wasp symbiont	KF843822 [46]
Ectropis obliqua virus	Geometrid tea moth	Lethal on larvae	AY365064 [62]
Graminella nigrifrons virus-1	Black-faced leafhopper	RNA seq	KP866792 [63]
Infectious flacherie virus	Mulberry silkworm	Larval dysentery/flacherie	AB000906 [64]
Ixodes scapularis iflavirus	Deer tick	RNA seq	LC094426 [65]
La Jolla virus	Fruit fly	RNA seq	KP714074 [66]
Lygus lineolaris iflavirus-1	Tarnished plant bug	Covert infection	JF720348 [67]
Nilaparvatea lugens honeydew virus-1	Brown planthopper	RNA seq	AB766259 [68]
Nilaparvatea lugens honeydew virus-3	Brown planthopper	RNA seq	AB826460 [69]
Perina nuda virus	Erebid moth	Flachery, minor pest of Ficus	AF323747 [70]
Perth bee virus-3	Honeybee	RNA seq	MG995731 [2]
Sacbrood virus	Honeybee	Failed larval moult, behaviour	AF092924 [53]<br>AF469603 [71]
Slow bee paralysis virus	Honeybee	Covert, paralysis with injection	EU035616 [50
Spodoptera exigua iflavirus-1	Beet armyworm	Covert, highly infectious	JN091707 [72]
Victoria bee virus-1	Honeybee	RNA seq	MG995723 [2]
Victoria bee virus-2	Honeybee	RNA seq	MG995724 [2]
